# Beyond the Surface: Novel Therapy Approach for Pancreatitis in the Setting of CFTR Dysfunction

**DOI:** 10.1155/crgm/9525069

**Published:** 2026-01-13

**Authors:** Brittany A. Wright, Ellie Hodapp, Douglas B. Hornick, Tahuanty A. Peña

**Affiliations:** ^1^ Department of Pharmaceutical Care, University of Iowa Health Care, Iowa City, Iowa, USA; ^2^ Department of Pharmacy Practice and Sciences, Skaggs School of Pharmacy and Pharmaceutical Sciences, University of California San Diego, La Jolla, California, USA, ucsd.edu; ^3^ Department of Internal Medicine, University of Iowa Health Care, Iowa City, Iowa, USA

**Keywords:** CFTR dysfunction, highly effective modulator, pancreatitis

## Abstract

**Objectives:**

Despite cystic fibrosis transmembrane conductance regulator (CFTR) proteins being present throughout the entire body and organ systems, typical presentation of cystic fibrosis (CF) involves lung disease. We report a series of individuals that were referred to our CF clinic for evaluation following a diagnosis of chronic pancreatitis or acute recurrent pancreatitis. After evaluation, they were deemed to have CFTR dysfunction and started on highly effective modulator therapy (HEMT).

**Methods:**

A retrospective chart review was completed for all individuals referred to the University of Iowa Health Care Cystic Fibrosis Clinic from January 1, 2012, through December 31, 2023, for follow‐up evaluation of chronic or acute recurrent pancreatitis. Along with demographic data, we quantified emergency department encounters or hospitalizations due to pancreatitis pre‐ and post‐HEMT as well as individual tolerability of therapy.

**Results:**

Ten individuals met inclusion criteria. Four individuals were found to have sweat chlorides that confirmed a diagnosis of CF, and three individuals with sweat chloride values below the diagnostic threshold were further evaluated with nasal potential difference (NPD) testing and found to have results consistent with CFTR dysfunction. All 10 patients were initiated on HEMT. In the 2 years prior to HEMT initiation, there was an average of 0.33 pancreatic episodes per month (range: 0.00–1.38), compared with 0.09 pancreatic episodes per month (range: 0.00–0.50) after initiation. HEMT was generally well tolerated with one individual discontinuing due to liver function abnormalities.

**Conclusions:**

Our study highlights a panel of individuals that may have been overlooked for CFTR dysfunction due to a nonpulmonary primary presentation, such as pancreatitis. However, our use of additional diagnostic tools followed by HEMT utilization was able to describe individual‐reported improvements in symptoms and quality of life in individuals with CFTR dysfunction.

## 1. Introduction

Cystic fibrosis (CF) currently impacts nearly 40,000 individuals in the United States and is classified as a condition resulting from impairment of the cystic fibrosis transmembrane conductance regulator (CFTR) protein [1]. The evaluation of CFTR dysfunction, including a diagnosis of CF, can be complex. The most recent guidelines suggest that a diagnosis of CF can be made in individuals presenting with a positive newborn screen, clinical features consistent with CF, or a positive family history if the sweat chloride value is ≥ 60 mmol/L [2]. Individuals with the aforementioned criteria but sweat chloride values in the intermediate range (30–59 mmol/L) on two separate occasions may have CF. It is generally recognized that additional CFTR gene analysis and/or CFTR functional analysis (nasal potential difference (NPD) or intestinal current measurements (ICMs)) should be completed. Lastly, individuals with clinical features that may be consistent with CF who have a sweat chloride < 30 mmol/L indicate that CF is less likely; however, it may be considered if evolving clinical criteria and/or CFTR genotyping support CF and not an alternative diagnosis, whereas a diagnosis of CFTR‐related disorder is described as a dysfunction of a singular system (i.e., pancreatitis and bronchiectasis) associated with CFTR dysfunction that does not fulfill the previously mentioned diagnostic criteria for CF.

The reported prevalence of CF carriers, individuals identified as having one CFTR variant, increases vastly with an impacted population nearing 10 million [3]. Historically, individuals with one known variant may have been told that they would be devoid of any clinical manifestations of disease. Recent data demonstrate that CFTR carriers are more likely to develop CF‐related conditions than a matched cohort, including pancreatitis [4, 5]. The European Cystic Fibrosis Society (ECFS) Diagnostic Network Working Group recently reported on the increased need for identification and care of seven different CFTR‐related disorders, including pancreatitis [6]. This evolving knowledge further underscores the need for timely identification of potentially CFTR‐related disorders and referral to an accredited CF center for further CFTR dysfunction testing.

In 2012, ivacaftor was the first approved medication in a class of therapies known as CFTR modulators, which improves the function of CFTR [7]. Of the several iterations approved since, the most effective and wide‐reaching is elexacaftor/tezacaftor/ivacaftor (ETI), approved in November 2019 [8, 9]. These therapies, collectively known as highly effective modulator therapies (HEMTs) have produced vast improvement in lung function, reduced hospitalizations and pulmonary exacerbations, and improved BMI, all combining for an enhanced quality of life [7–9]. Managing CFTR‐related conditions has remained challenging, likely due to under‐referral of these individuals. Two exceptions are the recent treatment with ivacaftor of a CF carrier with chronic pancreatitis, complicated by concomitant methylmalonic acidemia as well as an ongoing clinical trial utilizing ETI in individuals with non‐CF bronchiectasis [10, 11]. Given the significant healthcare utilization in acute and chronic pancreatitis [12], we reviewed individuals referred to our CF center for a primary presentation of pancreatitis, evaluated additional diagnostic modalities to determine if CFTR‐related dysfunction was present, and evaluated their response to HEMT initiation.

## 2. Materials and Methods

This study was submitted and approved by the University of Iowa Institutional Review Board. A retrospective chart review of the electronic health records (EHRs) at the University of Iowa Cystic Fibrosis Clinic was completed for all individuals seen January 1, 2012, through December 31, 2023, with a primary presentation of pancreatitis, following thorough workup and referral from our institutions’ Pancreatic Disorders Clinic. The following information, in addition to demographic data, was collected from the EHR pre‐ and post‐HEMT for all included individuals: weight, ppFEV1, encounters related to pancreatitis, need for pancreatic enzyme replacement therapy (PERT), sweat chloride testing, NPD results, pulmonary exacerbations, and associated need for oral or intravenous antibiotics. Pre‐HEMT data were collected for 2 years prior to HEMT initiation and included any emergency department encounter or hospitalization due to pancreatitis. Post‐HEMT data included any occurrences from medication initiation until December 31, 2023.

## 3. Results

Ten individuals met inclusion criteria and presented to our CF clinic following referral for evaluation of pancreatitis. Seven of these individuals had a single CFTR variant, while three had a second variant of varying clinical consequence (MVCC). Out of the nine individuals with sweat chloride results, four reached levels diagnostic of CF. NPD was performed in three individuals with nondiagnostic sweat chlorides, and all yielded results consistent with CFTR dysfunction. The remaining two pediatric individuals with nondiagnostic sweat chlorides did not undergo NPD. A full diagnostic testing history could not be located for one individual with two CFTR variants. Individual cases were clinically assessed by the provider at the time of the visit and diagnosed as CF or CFTR‐related disorder.

Out of the 10 individuals presented in this study, two were prescribed treatment with ivacaftor before transitioning to ETI. The other eight individuals were initially prescribed ETI. The age of HEMT initiation ranged from 6 years to 62 years with a larger percentage of female individuals (70%). Total time on HEMT at study conclusion ranged from 2 to 87 months, with the average time on therapy being 31.8 months. In the 2 years prior to HEMT initiation, there was an average of 0.33 pancreatic episodes per month (range: 0.00–1.38), compared with 0.09 pancreatic episodes per month (range: 0.00–0.50) after initiation. Subjectively, most individuals reported improvement in abdominal pain and gastrointestinal symptoms, consistent with the decreased hospitalization rates. There was no need for antibiotics to treat a pulmonary exacerbation in either the pre‐ or post‐HEMT periods with mean ppFEV1 98% prior to the start of HEMT, highlighting the absence of pulmonary symptoms in these individuals. Four individuals reported the use of PERT prior to HEMT initiation whereas five reported use after HEMT. Generally, HEMT was well tolerated in most individuals with some reports of changes to stools (i.e., diarrhea and constipation) and weight gain. Insomnia led to dose reduction in one individual and LFT elevation led to therapy discontinuation in another. For the eight individuals with data points available at 6 months, mean weight gain was 2.4 kg (range: −13.6–7.9 kg). At 12 months post‐HEMT, seven individuals reported weight gain (mean: 8.2 kg; range: −2.7–14.7 kg). These results are further summarized in Table 1.

**Table 1:** Individual experiences.

Variant(s)	Genetic test utilized	Sweat chloride completed and results if applicable (mmol/L)	NPD completed and results	Age at HEMT initiation (y)	Pre‐HEMT emergency/hospitalizations for pancreatitis (m)	Post‐HEMT emergency/hospitalizations for pancreatitis (m)	Total time on HEMT at the time of report (m)	Subjective reports after HEMT initiation	Adverse effects	HEMT dose modification
F508del	Ambry 508 First	Yes; 67, 61	No	37	0.58	0.25	12	Great improvement in symptoms	Insomnia	Yes; dose reduction
F508del	Ambry 508 First	Yes; 64, 63	No	62	0.04	0.05	43	Most abdominal pain resolved on ETI, still some cycles every 3‐4 weeks of RUQ pain	Constipation	No
F508del	Ambry CustomNext‐Cancer (full CFTR gene sequencing)	Yes; 15, 15	Yes; diagnostic	50	1.38	0.50	2		Rhinorrhea	No
G551D	Emory Warner Path Lab (87 mutation panel)	Yes; 60.8, 73.5	No	47	0.00	0.00	74	Improved GI tract symptoms and increased energy level	None	No
G551D	Ambry 508 First	Yes; 28, 18	Yes, diagnostic	40	0.50	0.13	8	Improvement since starting	Diarrhea	No
R117H	Ambry whole gene sequencing	Yes; 69, 78	No	29	0.29	0.00	20	Complete resolution of recurrent pancreatitis	None	No
R117H	Ambry whole gene sequencing	Yes; 39, 39	No	6	0.29	0.00	12		None	No
F508del/D1152H	Ambry 508 First	Yes; 41, 43	Yes, diagnostic	44	0.17	0.00	48	Pancreatic pain improved without the need of hospitalization	Weight gain	No
F508del/R117H	Mayo Genetics Lab (48 mutation panel)	Not available	No	37	0.00	0.00	87	Attributes persistent improvement to ETI; some pancreatitis events which were managed at home without admission	None	No
G551D/D1152H	NBS	Yes; 28, 24	No	15	0.08	0.00	12		LFT elevation	Yes; dose reduction and then therapy discontinuation

## 4. Discussion

The individuals described in this case series include several CFTR variant configurations. Following a gastrointestinal workup showing no other etiology for their pancreatitis, these individuals were referred to our clinic to assess for CFTR dysfunction as a potential etiology of their pancreatitis. As reported, CFTR dysfunction was present in all individuals that underwent additional testing. Overall, our population experienced a decrease in emergency department encounters and hospitalizations due to pancreatitis with limited adverse effects post‐HEMT initiation. While two individuals did require treatment modification or discontinuation, they still noted a decrease in episodes of pancreatitis after their treatment with HEMT, raising the question whether sustained full‐dose therapy would be necessary. PERT usage before and after HEMT initiation was difficult to interpret given the inconsistent retesting of fecal elastase and the confounding residual inflammation from pancreatitis.

In our experience, HEMT has been transformational in these patients, improving the quality of life as well as reducing the utilization of healthcare resources. However, we acknowledge that our study has several limitations. First, as a retrospective chart review, we concede challenging aspects to data collection including medication adherence and documentation inconsistencies. Second, as a small case series, we can provide observations but cannot draw large‐scale conclusions. Furthermore, the included individuals had varying lengths of therapy with some having short follow‐up timelines. This reduced duration does not preclude future episodes of pancreatitis; however, given the notable decrease, it seems likely that it would continue over a longer time course. Future studies could expand to longer timelines and other extrapulmonary outcomes such as sinus disease and aquagenic wrinkling. Quantification of CFTR function in individuals classified as carriers of CFTR variants could also shed light on the value of these therapies in additional populations. Given the substantial cost of HEMT and significant number of individuals that may be experiencing clinical effects of CFTR dysfunction, economic analysis will likely be justified.

## 5. Conclusion

Individuals with recurrent pancreatitis or other CFTR‐related conditions should be referred to an accredited CF center for broader evaluation, to identify CFTR variants, as well as quantify CFTR function via sweat chloride testing, NPD, or ICM. These evaluations are imperative to assess eligibility for CFTR modulators to relieve symptoms and problems related to CFTR dysfunction for many of these presenting phenotypes.

## Conflicts of Interest

The authors declare no conflicts of interest.

## Funding

No funding was received for this research.

## Data Availability

Research data are not shared.
